# Poor glycaemic control: prevalence, factors and implications for the care of patients with type 2 diabetes in Kinshasa, Democratic Republic of the Congo: a cross-sectional study

**DOI:** 10.3389/fcdhc.2023.1241882

**Published:** 2023-11-20

**Authors:** Jean-Pierre Fina Lubaki, Olufemi Babatunde Omole, Joel Msafiri Francis

**Affiliations:** ^1^ Department of Family Medicine and Primary Care, School of Clinical Medicine, Faculty of Health Sciences, University of the Witwatersrand, Johannesburg, South Africa; ^2^ Department of Family Medicine and Primary Care, Protestant University of Congo, Kinshasa, Democratic Republic of Congo

**Keywords:** diabetes mellitus, type 2, factors, glycaemic control, cross-sectional study, sub-Sahara Africa

## Abstract

**Introduction:**

Diabetes is a significant problem in sub-Saharan Africa and achieving glycaemic control poses a health challenge among patients living with type 2 diabetes. There are limited data on glycaemic control in Kinshasa, Democratic Republic of the Congo. This study assessed the prevalence and factors associated with glycaemic control to inform potential interventions to improve glycaemic control in Kinshasa.

**Methods:**

This was a cross-sectional study conducted between November 2021–September 2022 among patients recruited from 20 randomly selected health facilities in Kinshasa. Participants were asked to complete a structured questionnaire and to provide two millilitres of blood for Hb1AC assay. Poor glycaemic control was defined as HbA1c ≥7%. Univariate and multivariable logistic regressions were performed to identify factors associated with poor glycaemic control.

**Results:**

A total of 620 participants were recruited for this study. Study participants had a median age of 60 (IQR=53.5-69) years with the majority being female (66.1%), unemployed (67.8%), having income below the poverty line (76.4%), and without health insurance (92.1%). About two-thirds of the participants (420; 67.6%) had poor glycaemic control. Participants on monotherapy with insulin (AOR=1.64, 95%CI [1.10-2.45]) and those on a treatment duration ≥7 years (AOR=1.45, 95%CI [1.01-2.08]) were associated with increased odds of poor glycaemic control while being overweight (AOR= 0.47, 95%CI [0.26-0.85]) and those with uncontrolled blood pressure (AOR=0.65, 95% CI [0.48-0.90]) were protective for poor glycaemic control.

**Conclusion:**

Poor glycaemic control is prevalent among patients with type 2 diabetes in Kinshasa, DRC. Being on insulin alone and a duration of diabetes treatment equal or more than 7 years predisposed to poor glycaemic control. By contrary, having uncontrolled blood pressure and being overweight had protective effect against poor glycaemic control. These links between uncontrolled blood pressure and overweight on the one hand, and glycaemic control on the other are unusual. These reflect, among other things, the specific characteristics of diabetes in sub Saharan Africa.

## Introduction

Type 2 diabetes is increasing worldwide (1) – it is expected that the greatest increase in diabetes prevalence will take place in low- and middle-income countries (2). On the African Continent, type 2 diabetes is progressing rapidly due to modifiable risk factors, such as obesity and urbanisation (2).

In sub-Saharan Africa (SSA), diabetes care faces numerous challenges leading to unmet needs and a greater impact on morbidity and mortality (3, 4). In the Democratic Republic of the Congo (DRC), the prevalence of diabetes is estimated to be 5.8% for adults aged 20 to 79 years (4), with higher proportions of persons living with diabetes found in urban areas and the western part of the country (5, 6).

Good glycaemic control is the cornerstone of diabetes management, as it delays the onset of complications, reduces the cost of care and improves persons with diabetes quality of life. Nevertheless, the control of diabetes remains a challenge worldwide, with only about 50% of the person with diabetes controlled (7). In SSA, it is estimated that less than one-third of persons with type 2 diabetes achieve target glycaemic levels (8, 9). A recent systematic review of the studies on glycaemic control found: age, sex, poor socio-economic conditions, place of residence, positive family history of diabetes, longer duration of diabetes, treatment modalities and effects, alcohol consumption, smoking, presence of comorbidities or complications, and poor management were associated with poor glycaemic control (9). Contrarily, high diabetes health literacy, positive perception of family support, adequate coping strategies, dietary adherence, physical activity, adherence to follow−up appointments and medications, were associated with good glycaemic control (9).

An accurate knowledge of factors driving glycaemic control in a particular setting is essential to developing an intervention package to improve glycaemic control. Multiple factors drive glycaemic control in SSA, differing across settings (8). In the DRC, the prevalence of poor glycaemic control among persons with type 2 diabetes was reported as high as 86% and 79.9% have been reported in the nearby province of Kwilu and Kinshasa, respectively (10, 11). In these studies, Sagastume et al. (11) found that persons with diabetes older than 40 years of age had higher odds of achieving good glycaemic control than those younger while Blum et al. (10) found that abdominal obesity and having a body mass index (BMI) > 25 Kg/m2 were associated with poor glycaemic control. The study by Blum et al. (10) was conducted in a single site while Sagastume et al. (11) proceeded to a retrospective analysis of Kinshasa Primary HealthCare Network. Thus very few studies have been devoted to the factors of glycaemic control in DRC leading to a very rudimentary data on the issue and poor understanding of glycaemic control. In anticipation of the building of an intervention package to deal with the issue in Kinshasa, and in an effort to expand knowledge about blood glucose control factors, we designed a mixed-method cross-sectional study. Studies with mixed methodology are appropriate to explore complex phenomena in a broad way. In this article, we present the results of the quantitative phase.

## Materials and methods

### Study design

This was a cross sectional study, a component of a bigger research project on glycaemic control among persons with type 2 diabetes in Kinshasa, DRC, for which the study protocol was previously published (12). Routinely, type 2 diabetes is defined by a bundle of epidemiological and clinical arguments: onset of diabetes at more than 40 years, history of diabetes in the family, presence of autoimmune pathology, association with metabolic syndrome: hypertension, upper BMI, dyslipidaemia, presence of ketone bodies and favourable response of treatment to oral hypoglycaemic agents (OHA).

### Study setting

Our study was multisite within Kinshasa, a city of about 15 million inhabitants spread over an area of 9,965 km^2^ (13). The participants were recruited from 20 randomly selected health facilities in Kinshasa, DRC. The study was conducted in the health facilities belonging to the Catholic Church and the Salvation Army. With a total of 66 health facilities (1 referral hospital and 65 health centres) distributed across 24 health districts, these organisations own most of the facilities that have integrated diabetes care in primary care in Kinshasa.

### Study population

The study population consisted of persons with type 2 diabetes attending health centres that offer diabetes care in the Kinshasa Primary Care Network, with about 7326 persons with diabetes registered in 2020. The inclusion criteria were age ≥18 years, receiving diabetes treatment for at least six months and consenting to the study. The exclusion criteria were pregnancy, and having difficulty communicating due to mental disability.

### Sample size estimation

The estimated minimum sample size was computed using Epi info version 7.2.2.2. Assuming that the prevalence of poor glycaemic control was 68% (14), a 95% confidence level and a power of 80%, 59,2% of persons with diabetes who had a diabetes duration ≤7 years (unexposed) presented with poor glycaemic control, and 74,4% of those who had a diabetes duration >7 years (exposed) presented with poor glycaemic control (15). The unexposed to exposed ratio is 0.47 (15). The minimum estimated sample size was 368. Adjusting for a design effect of 1.5, the calculated sample size of 552 was determined. To account for an estimated 10% non-response rate, the minimum required sample size was 614 persons with diabetes, rounded up to 620.

### Sampling of participants

Participant selection was a two-stage process. The first stage was the random selection of 20 out of 48 healthcare facilities. As the healthcare facilities have an unequal number of persons with diabetes, the participants were selected by probability proportional to the patient population size. The second stage consisted of the selection of the participants; 31 patients were selected from each selected healthcare facility using systematic sampling. The research assistant was taking the record of the first patient on a clinic day to assess eligibility and subsequently was taking the record of every third patient for questionnaire administration. If the first patient was not eligible or if the third patient selected in the next step was not eligible, the research assistant selected the next patient(s) until an eligible patient was obtained, then continued with the selection of each third patient. This process ensured each patient had the same probability of selection.

### Data collection

The data collection process lasted from November 2021–September 2022. For each participant, the research team performed physical and anthropometric measurements. These measurements were taken once by trained staff members on the same portable equipment at all the health facilities. The questionnaire consisted of pre-existing standardised tools translated from English into French and Lingala. The questionnaire was pre-tested before data collection, no changes were necessary on the tools for use in our study. At the end of the interview, 2 millilitres of venous blood was collected in a tube with EDTA from the participant. The tube was identified and put in a fridge at 2-8 °C—when the centre has a fridge—or directly in the isotherm box prepared. At the end of the visit, all the samples were transferred in the laboratory using the isotherm box. The questionnaire was administered using REDCap (Research Electronic Data Capture) on a tablet or smartphone (16). Information captured during the interview on the history of diabetes was verified with what is recorded in the medical records.

### Variables of the study

#### Outcome

The main outcome variable was poor glycaemic control, defined as HbA1c ≥7% (17–19), and obtained from the blood sample assayed at the laboratory of the School of Medicine at the Protestant University of Congo in Kinshasa. The assay was performed using an automated Genuis WP 21B with antibody-based immunoassay method of Cypress Diagnostics (20).

#### Exposures

The possible determinants for glycaemic control considered in this study were sociodemographic parameters (age, sex, marital status, educational attainment, occupation, income, use of health insurance, access to food, distance from place of residence to health centre), lifestyle parameters (smoking, problematic alcohol consumption), clinical parameters (duration of diabetes, height, weight, body mass index, waist circumference, presence of comorbidities, blood pressure, treatment, duration of treatment), and psychological parameters (adherence to treatment, depression, diabetes distress, social support, self-management, knowledge). **Supplementary file 1** detailed the exposures, their measurements, operational definitions, reliability, and references.

### Data analysis

All the analyses were performed using survey data analysis with STATA 17 (21) to account for the study design characteristics. We expressed age as median with interquartile range (IQR), as it was not normally distributed. The other variables were analysed as categorical variables and expressed as frequency (n) and percentage (%). Bivariate analysis was performed to compare uncontrolled versus controlled participants in terms of glycosylated haemoglobin using the Chi-square/Fisher exact test for categorical variables. We further carried out multivariable logistic regression to assess factors associated with glycaemic control. Age, sex, duration of treatment, and food security were included in the regression model a priori. Other variables with a p-value <0.2 in univariate analysis were also included in the model. The p-value of <0.05 was considered statistically significant.

## Results

A total of 620 participants were included in the study out of a total of 627 invited, accounting for a non-response rate of 1.1%. The participants had a mean age of 60.21 ± 12.44 years, a mean BMI of 23.60 ± 5.21 Kg/m^2^, a mean waist circumference of 89.95 ± 13.49 centimetres, a mean duration of diabetes disease of 83.17 ± 75.18 months, and a mean duration of 82.17 ± 75.02 months. **Table 1** summarises the sociodemographic, lifestyle and clinical characteristics of the participants. Fewer than two-thirds of the participants (64.68%) of the participants presented a complication or a comorbidity. The most common complications or comorbidities were hypertension (53.88%), diabetic retinopathy (15.79%), erectile dysfunction (14.04%), and cataract (3.51%). A slight more than half of the participants (53.87%) were on insulin alone while 38.23% and 7.9% of the participants on Oral Hypoglycaemic agents (OHA) and Mixed treatment (Insulin-OHA). The most common OHA were: Metformin (69.29%), Glibenclamide (15.04%), Gliclazide (11.04%), and Glimepiride (3.15%).

**Table 1:** Sociodemographic characteristics of participants with type 2 diabetes in Kinshasa, n=620 (2021-2022).

	N (%)
Age (years)	
** 18-39**	39 (6.4)
** 40-64**	342 (55.1)
** ≥ 65**	239 (38.5)
Sex	
** Male**	211 (33.9)
** Female**	409 (66.1)
Marital status	
** Single**	199 (32.2)
** Married**	393 (63.3)
** Other**	28 (4.5)
Educational level	
** No formal education**	77 (12.5)
** Primary school**	199 (32.1)
** Secondary school**	269 (43.4)
** Tertiary**	75 (12.1)
Occupation	
** Employed**	132 (21.1)
** Unemployed**	419 (67.7)
** Other**	69 (11.2)
Income	
** Below the poverty line**	473 (76.4)
** Above the poverty line**	147 (23.6)
Access to food	
** Food security**	249 (40.0)
** Mildly Food Insecure Access**	77 (12.5)
** Moderately Food Insecure Access**	107 (17.3)
** Severely Food Insecure Access**	187 (30.2)
Health insurance	
** Insured**	49(7.9)
** Without health insurance**	571 (92.1)
Distance to a health facility	
** Nearby**	451 (72.6)
** Distant**	165 (27.4)

### Prevalence of glycaemic control and participants’ characteristics

About two-thirds of the participants (67.8%; n=420) had poor glycaemic control.

There was no statistically significant difference between controlled and uncontrolled participants in terms of sociodemographic characteristics (**Table 2**).

However, controlled participants differed significantly from uncontrolled participants in terms of BMI (p=0.005), control of blood pressure (p=0.027), and treatment regimens (p=0.002) (**Table 2**).

**Table 2:** Sociodemographic, lifestyle and clinical characteristics in relation with glycaemic control among participants with type 2 diabetes in Kinshasa, n=620 (2021-2022).

	All participants (n, %)	Good glycaemic control (n, %(95%CI))	Poor glycaemic control (n, %(95%CI))	p
Sociodemographic characteristics				
Age				0.479
18-39	39 (100.0)	9 (22.8 (10.7-42.2))	30 (77.2 (57.8-89.3))	
40-64	342 (100.0)	112 (32.7 (26.7-39.4))	230 (67.3 (60.6-73.3))	
≥ 65	239 (100.0)	79 (33.0 (24.8-42.2))	160 (67.0 (57.6-75.2))	
Sex				0.989
Male	211 (100.0)	68 (32.2 (24.6-40.8))	143 (67.8 (59.2-75.4))	
Female	409 (100.0)	132 (32.2 (25.3-40.0))	277 (67.8 (60.0-74.7))	
Marital status				0.413
Single	199 (100.0)	68 (34.3 (25.7-43.9))	131 (65.7 (56.1-74.3))	
Married	393 (100.0)	126 (31.3 (25.4-39.3))	267 (65.7 (60.7-74.6))	
Other	28 (100.0)	6 (21.3 (9.7-40.6))	22 (78.7 (59.4-90.3))	
Educational status				0.781
No formal	77 (100.0)	28 (36.4 (18.2-59.7))	49 (63.6 (40.3-81.8))	
Primary school	199 (100.0)	64 (32.1 (24.6-40.6))	135 (67.9 (59.4-75.4))	
Secondary school	269 (100.0)	87 (32.3 (25.9-39.4))	182 (67.7 (60.6-74.1))	
University	75 (100.0)	21 (27.9 (19.5-38.3))	54 (72.1 (61.7-80.5))	
Occupation				0.518
Employed	132 (100.0)	45 (34.1 (27.2-41.9))	87 (65.9 (58.1-72.8))	
Unemployed	419 (100.0)	130 (30.9 (24.1-38.8))	289 (69.1(61.2-75.9))	
Other	69 (100.0)	25 (36.3 (25.6-48.6))	44 (63.7 (51.4-74.4))	
Income				0.342
Below the poverty line	473 (100.0)	149 (31.4 (25.1-38.5))	324 (68.6 (61.5-74.9))	
Above the poverty line	147 (100.0)	51 (34.7 (27.8-42.4))	96 (65.3 (57.6-72.2))	
Access to food				0.192
Food secure	249 (100.0)	69 (27.6 (19.7-37.3))	180 (72.4 (62.7-80.3))	
Mildly Food Insecure	71 (100.0)	28 (36.5 (24.2-50.9))	49 (63.5 (49.1-75.8))	
Moderately Food Insecure	107 (100.0)	43 (40.2 (31.5-49.6))	64 (59.8 (50.4-68.5))	
Severely Food Insecure	187 (100.0)	60 (31.9 (24.0-41.1))	127 (68.1 (58.9-76.0))	
Health insurance				0.782
Insured	48 (100.0)	17 (34.5 (20.2-52.2))	32 (65.5 (47.8-79.8))	
Uninsured	571 (100.0)	183 (32.0 (25.8-39.0))	388 (68.0 (61.0-74.2))	
Distance to a health facility				0.644
Nearby	451(100.0)	148 (32.8 (25.8-40.6))	303 (67.2 (59.4-74.2))	
Distant	169 (100.0)	52 (30.7 (23.7-38.7))	117(69.3 (61.3-76.3))	
Lifestyle characteristics				
Smoking				0.268
No	616 (100.0)	200 (32.4 (26.5-38.9))	416 (67.6 (61.1-73.5))	
Yes	4 (100.0)	0 (0.0 (0.0))	4 (100.0 (0.0))	
Alcohol disorder				0.566
No disorder	611 (100.0)	198 (32.4 (26.5-38.8))	413 (67.6 (61.2-73.5))	
Health risk consumption	9 (100.0)	2 (22.1 (4.0-66.2))	7 (77.9 (33.8-96.0))	
Clinical characteristics				
Duration of diabetes				0.549
0-5 years	267 (100.0)	84 (31.3 (23.0-41.0))	183 (68.7 (59.0-77.0))	
5-10 years	217 (100.0)	76 (35.1 (26.5-44.8))	141 (64.9 (55.2-73.5))	
≥10 years	136 (100.0)	40 (29.3 (22.2-37.5))	96 (70.7 (62.5-77.8))	
Body mass index				0.005*
Underweight	65 (100.0)	14 (21.6 (13.4-33.0))	51 (78.4 (67.0-86.6))	
Normal	347 (100.0)	102 (29.3 (21.9-37.9))	245 (70.7 (62.1-78.1))	
Overweight	142 (100.0)	66 (46.6 (39.1-54.2))	76 (53.4 (45.8-60.9))	
Obesity	66 (100.0)	18 (27.3 (15.7-43.1))	48 (72.7 (56.9-84.3))	
Waist circumference				0.083
Abnormal	243 (100.0)	93 (38.2 (29.8-47.3))	150 (61.8 (52.7-70.2))	
Normal	377 (100.0)	107 (28.3 (21.5-36.3))	270 (71.7 (63.7-78.5))	
Presence of comorbidities				0.266
Yes	401 (100.0)	136 (33.9 (27.6-40.7))	265 (66.1 (59.3-72.4))	
No	218 (100.0)	64 (29.2 (21.6-38.1))	155 (70.8 (61.9-78.4))	
Blood pressure				0.027*
Controlled	400 (100.0)	117 (29.2 (22.9-36.4))	283 (70.8 (63.6-77.1))	
Uncontrolled	220 (100.0)	83 (37.7 (30.8-45.2))	137 (62.3 (54.8-69.2))	
Treatment				0.002*
Insulin	334 (100.0)	87 (26.0 (20.7-32.1))	247 (74.0 (67.9-79.3))	
Oral hypoglycaemic agents	237 (100.0)	98 (41.3 (31.6-51.7))	139 (58.7 (48.3-68.4))	
Mixed	49 (100.0)	15 (30.7 (21.1-42.3))	34 (69.3 (57.7-78.9))	
Duration of treatment				0.229
< 7 years	387 (100.0)	132 (34.1 (27.3-41.6))	255 (65.9 (58.4-72.7))	
≥ 7 years	233 (100.0)	68 (29.1 (21.9-37.4))	165 (70.9 (62.6-78.1))	

*p<0.05.

Perceived support from significant others (p=0.005), perceived family support (p=0.020), treatment regimen distress (p=0.029), and adherence to physical activity (p=0.017) were significantly different between controlled and uncontrolled participants (**Table 3**).

**Table 3:** Psychological characteristics and glycaemic control among participants with type 2 diabetes in Kinshasa (n=620).

	All participants (n, %)	Good glycaemic control (n, %(95%CI))	Poor glycaemic control (n, %(95%CI))	p
Adherence to treatment				
High	351 (100.0)	110 (31.3 (23.4-40.3))	241 (68.7 (59.7-76.6))	0.427
Moderate	217 (100.0)	76 (35.0 (29.2-41.4))	141 (65.0 (58.6-70.8))	
Low	52 (100.0)	14 (26.8 (17.0-39.7))	38 (73.2 (60.3-83.0))	
Depression				
Without depression	612 (100.0)	198 (32.3 (26.5-38.7))	414 (67.6 (61.3-73.5))	0.535
Moderate depression	6 (100.0)	1 (16.7 (2.6-60.0))	5 (83.3 (40.0-95.4))	
Moderately severe depression	2 (100.0)	1 (50.1 (4.6-95.4))	1 (49.9 (4.6-95.4))	
Multidimensional perceived social support				
Total score				0.299
Low support	139 (100.0)	40 (28.8 (20.6-38.6))	99 (71.2 (61.4-79.4))	
Moderate support	460 (100.0)	150 (32.5 (25.6-40.4))	310 (67.5 (59.6-74.4))	
High support	21 (100.0)	10 (47.4 (27.8-67.8))	11 (52.6 (32.2-72.2))	
Significant others				0.006*
Low support	182 (100.0)	49 (26.9 (19.3-36.1))	133 (73.1 (63.9-80.7))	
Moderate support	325 (100.0)	98 (30.2 (23.2-38.2))	227 (69.8 (61.8-76.8))	
High support	113 (100.0)	53 (46.7 (38.1-55.6))	60 (53.3 (44.4-61.9))	
Family				0.020*
Low support	140 (100.0)	44 (31.5 (24.7-39.3))	96 (68.5 (60.7-75.3))	
Moderate support	386 (100.0)	111 (28.7 (21.4-37.2))	275 (71.3 (62.8-78.6))	
High support	94 (100.0)	45 (47.8 (36.3-59.5))	49 (52.2 (40.5-63.7))	
Friends				0.463
Low support	282 (100.0)	100 (35.3 (27.1-44.5))	182 (64.7 (55.5-72.9))	
Moderate support	321 (100.0)	95 (29.6 (20.7-40.4))	226 (70.4 (59.6-79.3))	
High support	17 (100.0)	5 (29.4 (17.1-45.6))	12 (70.6 (54.4-82.9))	
Diabetes distress				
Total score				0.113
No distress	268 (100.0)	105 (39.0 (31.8-46.8))	163 (61.0 (53.2-68.2))	
Moderate distress	138 (100.0)	37 (26.8 (19.5-35.5))	101 (73.2 (64.5-80.5))	
High distress	214 (100.0)	58 (27.2 (16.9-40.6))	156 (72.8 (59.4-83.1))	
Emotional				0.375
No distress	395 (100.0)	135 (34.1 (27.9-40.9))	260 (65.9 (59.1-72.1))	
High distress	225 (100.0)	65 (28.9 (19.8-40.2))	160 (71.1 (59.8-80.2))	
Regimen				0.033*
No distress	256 (100.0)	102 (39.7 (32.1-47.9))	154 (60.3 (52.1-67.9))	
Moderate distress	144 (100.0)	31 (21.5 (14.7-30.3))	113 (78.5 (69.7-85.3))	
High distress	220 (100.0)	67 (30.5 (20.7-42.5))	153 (69.5 (57.5-79.3))	
Interpersonal				0.293
No distress	247 (100.0)	91 (36.7 (29.1-45.1))	156 (63.3 (54.9-70.9))	
Moderate distress	130 (100.0)	37 (28.4 (22.3-35.4))	93 (71.6 (64.6-77.7))	
High distress	243 (100.0)	72 (29.6 (19.9-41.6))	169 (70.4 (58.4-80.1))	
Physician				0.134
No distress	278 (100.0)	106 (38.0 (30.8-45.8))	172 (62.0 (54.2-69.2))	
Moderate distress	129 (100.0)	40 (30.9 (25.7-36.6))	89 (69.1 (63.4-74.3))	
High distress	213 (100.0)	54 (25.5 (15.3-39.2))	159 (74.5 (60.8-84.7))	
Self-management				
Total score				0.748
Good adherence	595 (100.0)	193 (32.4 (26.2-39.3))	402 (67.6 (60.7-73.8))	
Poor adherence	25 (100.0)	7 (28.3 (11.2-55.3))	18 (71.7 (44.7-88.8))	
Dietary control				0.386
Good adherence	526 (100.0)	174 (33.0 (26.0-40.8))	352 (67.0 (59.2-74.0))	
Poor adherence	94 (100.0)	26 (27.7 (19.8-37.2))	68 (72.3 (62.8-80.2))	
Glucose management				0.511
Good adherence	552 (100.0)	181 (32.7 (26.5-39.6))	371 (67.3 (60.4-73.5))	
Poor adherence	68 (100.0)	19 (28.0 (16.4-43.4))	49 (72.0 (56.6-83.6))	
Physician contact				0.304
Good adherence	552 (100.0)	183 (33.1 (26.3-40.6))	369 (66.9 (59.4-73.7))	
Poor adherence	68 (100.0)	17 (25.1 (15.1-38.7))	51 (74.9 (61.3-84.9))	
Physical activity				0.019*
Good adherence	385 (100.0)	110 (28.5 (22.7-35.2))	275 (71.5 (64.8-77.3))	
Poor adherence	235 (100.0)	90 (38.2 (30.5-46.6))	145 (61.8 (53.4-69.5))	
Knowledge on diabetes				0.097
Low	174 (100.0)	62 (35.6 (27.6-44.6))	112 (64.4 (55.4-72.4))	
Acceptable	245 (100.0)	68 (27.7 (20.9-35.7))	177 (72.3 (64.3-79.1))	
Good	201 (100.0)	70 (34.7 (28.5-41.5))	131 (65.3 (58.5-71.5))	

*p<0.05.

### Determinants of glycaemic control

Being on monotherapy with insulin (AOR=1.64, 95%CI [1.10-2.45]) and having a treatment duration ≥7 years (AOR=1.45, 95%CI [1.01-2.08]) increased the odds of poor glycaemic control. On the other hand, being overweight (AOR= 0.47, 95%CI [0.26-0.85]) and having uncontrolled blood pressure (AOR=0.65, 95% CI [0.48-0.91]) decreased the odds of poor glycaemic control (**Table 4**). An analysis of the relation between BMI and poor glycaemic control in two separated groups as non-insulin and insulin users found that only in non-insulin users, being overweight was protective against poor glycaemic control ((AOR: 0.28,95%CI [0.10-0.80],p:0.020) versus (AOR:0.55, 95%CI [0.25-1.17], p:0.115) for non-insulin users and insulin users respectively).

**Table 4:** Results of survey logistic regression estimating the odds for poor glycaemic control in Kinshasa, 2021-2022.

	Univariate analysis		Multivariate analysis	
	p	COR (95%CI)	p	AOR (95%CI)
Age (years)				
18-39		1		1
40-64	0.276	0.61 (0.24-1.54)	0.652	0.81 (0.32 -2.08)
≥ 65	0.271	0.60 (0.23-1.54)	0.736	0.86 (0.35-2.13)
Sex				
Male		1		1
Female	0.989	1.00 (0.64-1.55)	0.494	1.16 (0.75-1.79)
Access to food				
Food secure		1		1
Mildly Food Insecure	0.199	0.66 (0.35-1.27)	0.079	0.59 (0.33-1.07)
Moderately Food Insecure	0.008	0.57 (0.38-0.85)	0.083	0.69 (0.46-1.05)
Severely Food Insecure	0.468	0.81 (0.45-1.46)	0.670	0.90 (0.53-1.51)
Body mass index				
Underweight		1		1
Normal	0.178	0.66 (0.36-1.22)	0.288	0.73 (0.41-1.32)
Overweight	0.000	0.32 (0.18-0.55)	0.015*	0.47 (0.26-0.85)
Obese	0.515	0.73 (0.28-1.94)	0.574	1.21 (0.60-2.48)
Waist circumference				
Normal		1		1
Abnormal	0.084	0.64 (0.38-1.07)	0.328	0.80 (0.51-1.26)
Blood pressure				
Controlled		1		
Uncontrolled	0.027	0.68 (0.84-0.95)	0.011*	0.65 (0.48-0.90)
Duration of treatment (years)				
< 7		1		1
≥ 7	0.229	1.26 (0.85-1.87)	0.043*	1.45 (1.01-2.08)
Treatment regimen				
OHA		1		1
Insulin	0.003	2.00 (1.31-3.05)	0.019*	1.10 (1.10-2.45)
Mixed	0.144	1.59 (0.84-2.99)	0.319	1.39 (0.71-2.74)
Significant other support				
Low support		1		1
Moderate support	0.465	0.85 (0.54-1.34)	0.244	0.78 (0.51-1.20)
High support	0.002	0.42 (0.25-0.71)	0.128	0.64 (0.36-1.15)
Family support				
Low support		1		1
Moderate support	0.586	1.14 (0.69-1.91)	0.533	1.22 (0.63-2.34)
High support	0.015	0.50 (0.29-0.86)	0.359	0.70 (0.31-1.55)
Diabetes distress				
No distress		1		1
Moderate distress	0.017	1.75 (1.12-2.73)	0.179	1.42 (0.84-2.39)
High distress	0.009	1.72 (0.83-3.54)	0.901	0.95 (0.42-2.23)
Physical activity				
Poor adherence		1		1
Good adherence	0.019	1.55 (1.08-2.22)	0.067	1.34 (0.98-1.80)
Knowledge on diabetes				
Low		1		1
Acceptable	0.004	1.45 (1.14-1.83)	0.054	0.69 (0.48-1.01)
Good	0.846	1.04 (0.68-1.59)	0.419	1.13 (0.83-1.52)

*p<0.05.

## Discussion

This study was designed to assess the extent of poor glycaemic control among persons living with type 2 diabetes in Kinshasa, including its driving factors. The study found that poor glycaemic control is very prevalent (67.8%) and no sociodemographic or lifestyle characteristics were associated with glycaemic control. While monotherapy with insulin and having a treatment duration ≥7 years increased the odds of poor glycaemic control, being overweight and having uncontrolled blood pressure reduced its odds.

That more than two-thirds of the study participants have poor glycaemic control corroborates the findings of other studies in SSA (8, 9). Furthermore, this study found a lower prevalence of poor glycaemic control than that found by Blum et al. in the nearby rural province of Kwilu in the DRC. In rural areas in the Democratic Republic of Congo, there are fewer centers that offer care to persons with diabetes, and the access to medicines and the range of foods are limited. Health system planners must work to ensure equitable geographical distribution of diabetes care centers, and regular and extensive supply of medications. Glycaemic control in our study was poorer than that found in the European or North American studies (22, 23), and indicated poor diabetes care in Kinshasa. Our result also translate the issues in SSA in general, where diabetes care faces multiple barriers, such as lack of funding for non-communicable diseases, lack of accurate guidelines directed to the specificities of diabetes in the population, lack of availability of medications and other supplies, and inequity between public and private sector diabetes care (3, 24). Moreover, self-management in SSA is poor and represents a threat to the health of individuals and capacity of the health system (25). An effective preparation of the health care systems to face diabetes burden is crucial and including effective financing, training of the healthcare providers, and provision of required materials and medicines (3, 26).

In this study, participants on monotherapy with insulin were 1,64 times more likely to have poor glycaemic control than those on oral hypoglycaemic drugs. Studies have shown that only around one-fourth of persons with diabetes on insulin could achieve glycaemic targets because they might be erroneously taking an insufficient daily dose and incorrectly titrating insulin (27). One may also hypothesize that as most of the persons with diabetes were unemployed and not covered by health insurance, they could have been unable to adequately follow the prescribed regimen when they lack money to pay for their medicines or food. The psychological resistance to insulin, prevalent in our setting according to the study by Rita et al. (28), could also be another explanation for poor glycaemic control among persons with type 2 diabetes in our study. In the diabetes attitudes, wishes and needs second study (DAWN), participants reported low confidence in the efficacy of insulin, with 26.9% of participants abstaining from insulin because they thought insulin unfeasible or impracticable to manage their diabetes (29). Healthcare providers must ensure that psychological resistance to initiating insulin is adequately addressed, and effectively train the persons with diabetes to correctly follow their prescriptions.

Type 2 diabetes is a lifestyle disease, and all guidelines recommend that insulin therapy should accompany lifestyle modification and oral hypoglycaemic drugs. The propensity of clinicians to use insulin may reflect the lack of appropriate guidelines or poor clinicians’ adherence to evidence-based clinical guidelines. There is also no system of safeguards to regulate medical prescriptions, especially since these are mostly provided in private pharmacies. Clear management guidelines must also be adapted for the use of available medicines and efforts must be made to offer new hypoglycaemic agents at affordable prices. The technical supervision of health facilities by the national programme is crucial to ensure the proper management of diabetes in accordance with standards.

In this study, the odds of overweight participants having poor glycaemic control were reduced by 53.0%. The sub-analysis also found that being overweight was protective only for non-insulin users. Our finding contrasted the well-known relationship between being overweight and suboptimal glycaemic control and poor glycaemic control (30). One may note that in our context, poor glycaemic patients are receiving, in most instances, insulin. Once they are better controlled, they are put on OHA. Thus, it is possible that current patients receiving OHA gained weight during a previous treatment with insulin phase and are beginning oral treatment with better glycaemic control. Blum et al. (10), in their study near Kinshasa, also found that BMI>25 Kg/m^2^ and abdominal obesity were protective against poor glycaemic control. The authors stated that this finding could reflect the existence of special features of diabetes in SSA. Weight loss or the prevention of weight gain is an important goal in the management of type 2 diabetes or prediabetes (31). However, increasing weight could also arise in persons with diabetes due to the effect of antidiabetic medication on body weight. Apart from metformin and thiazolidinediones, other antidiabetic agents could lead to weight gain (32). In Kinshasa, insulin is largely used and there has been a limited range of affordable medications for persons with diabetes. Healthcare providers must furthermore ensure that the persons with diabetes are adequately managed to avoid adverse effects (32).

In the study sample, persons with diabetes having uncontrolled blood pressure reduced the odds of having poor glycaemic control by 35.0%. Mobula et al. (33), in a Ghanaian study, also found that systolic blood pressure was significantly higher among persons with diabetes having adequate glycaemic control compared to the group with poor glycaemic control. As discussed by Mobula et al. (33), among persons with diabetes having good glycaemic control, it can be that there was a significantly higher proportion of patients with dual diagnosis—hypertension and diabetes. This observation can also be explained by the fact that health providers give more attention to persons with comorbidity or an increase in healthcare utilisation by the persons with comorbidity (34). Hypertension is frequently associated with diabetes (35), which indicates that more insight into adequate management of hypertension among persons with diabetes in our setting will be required (34).

This study found that a treatment duration ≥7 years increased the odds of poor glycaemic control by 1,45 times. Longer duration of treatment has been linked to poor glycaemic control in SSA (36). As diabetes is a progressive disease with deterioration in the function of the ßeta cells of the pancreas with time, more adjustments in the treatments are required in older persons with diabetes who would generally have had diabetes longer and are more likely to have comorbidities (37). Health providers must be informed of the progression of diabetes and be able to adjust the treatments for persons with diabetes accordingly.

Most of our participants (93.7%) were older than 40 years. This proportion aligns with the classic description of type 2 diabetes, in which the disease appears in individuals older than 40 years most of the time. Female persons with diabetes represented approximately two-thirds of the participants. A retrospective analysis of the Kinshasa Health Network database conducted by Sagastume et al. (11) also found the same-sex prevalence. This high prevalence of type 2 diabetes affects more women than men, due to the higher metabolic risk in the former (38). Furthermore, the health-seeking behaviour of women is better than in men (39). Most of the participants were unemployed, poor and without health insurance, which has been representative of the condition of the general population in Kinshasa. No sociodemographic and lifestyle characteristics were associated with poor glycaemic control. Our finding here has been corroborated by the study of Blum et al. (10), who also found no sociodemographic or lifestyle factors associated with poor glycaemic control in a cross-sectional survey in the nearby province of Bandundu in the DRC. However, we can discuss the efficiency of the assessment of certain characteristics such as income in our environment. The income assessed was the individual’s income and did not take into account the contribution of relatives, which was sometimes substantial. And since most persons with diabetes were not paid employees, those in the informal or liberal sector could not accurately determine their income. In a retrospective study in Kinshasa, Sagastume et al. (11) found that younger persons with diabetes needed prioritised attention to reach glycaemic targets. Nevertheless, interventions for better glycaemic control have to prioritise vulnerable groups, such as younger and older age, women and non-insured persons with diabetes (9). Implementing universal coverage can increase access to care for the aforementioned groups (40).

### Limitations of the study

This study estimated the extent of poor glycaemic control among persons with type 2 diabetes in Kinshasa. Because of the cross-sectional nature of the study, it is not possible to ascertain a causal relationship between poor glycaemic control and the determinants. Other potential biases include selection bias as only persons with diabetes who attended the diabetic clinics in the period of the study could be included in the study, recall bias as for some responses, the participants might refer to their history with the possibility to have lost memory or omitted details for some events, interviewer bias since the data collectors could have influenced the participants by how they asked questions or reacted to the answers, and social desirability bias since participants could have given answers that made them look good to respondents and did not talk about their true experiences (41). These biases were minimised by ensuring effective training of the data collectors to make certain that the aim and objectives of the study were clearly stated to the participants, and that questions were asked in a non-judgemental way.

Nonetheless, this study provides an understanding of important factors on which to focus for improved glycaemic control in Kinshasa, DRC or similar settings, particularly in sub-Saharan Africa.

## Conclusion

Poor glycaemic control is prevalent among persons with type 2 diabetes in Kinshasa, DRC. Being on insulin alone and a duration of diabetes treatment equal or more than 7 years predisposed to poor glycaemic control. By contrary, having uncontrolled blood pressure and being overweight had protective effect against poor glycaemic control. These links between uncontrolled blood pressure and overweight on the one hand, and glycaemic control on the other are unusual. These reflect, among other things, the specific characteristics of diabetes in sub-Saharan Africa.

## Availability of data and materials

All data generated or analysed during this study are included in this published article and its **Supplementary Information Files**.

## Ethics approval

The researchers declared that they complied with the conditions under which this study obtained approval from the ethics committees of the Protestant University of Congo (reference number: CEUPC 0067; Date: 05/02/2021) and Human Research Ethics Committee (Medical) of the University of the Witwatersrand (reference number: M210308; Date: 26/08/2021). The study was conducted according to the ethical guidelines of the Declaration of Helsinki. Permission was obtained from the Kinshasa Primary HealthCare Network to conduct the study. Informed consent was obtained from each participant. Data collection was done in strict adherence to local COVID-19 regulations.

## Data availability statement

The original contributions presented in the study are included in the article/**Supplementary Materials**, further inquiries can be directed to the corresponding author.

## Ethics statement

The studies involving humans were approved by Ethics committee of the Protestant University of Congo (reference number: CEUPC 0067; Date: 05/02/2021) and Human Research Ethics Committee (Medical) of the University of the Witwatersrand (reference number: M210308; Date: 26/08/2021). The studies were conducted in accordance with the local legislation and institutional requirements. The participants provided their written informed consent to participate in this study.

## Author contributions

J-PL, OO, and JF designed the study. J-PL contributed to acquisition of funding and data. J-PL oversaw the research process. J-PL and JF performed the data processing and quality control. J-PL conducted the statistical analyses, drafted the manuscript, and is the guarantor of this work. J-PL, OO, and JF interpreted the data. All authors contributed to the article and approved the submitted version.

## Supplementary material

Supplementary file 2 xls — Study dataset. Anonymised database.
